# Short Sleep Duration by Occupation Group — 29 States, 2013–2014

**DOI:** 10.15585/mmwr.mm6608a2

**Published:** 2017-03-03

**Authors:** Taylor M. Shockey, Anne G. Wheaton

**Affiliations:** ^1^Division of Surveillance, Hazard Evaluations, and Field Studies, National Institute for Occupational Safety and Health, CDC; ^2^Division of Population Health, National Center for Chronic Disease Prevention and Health Promotion, CDC.

The American Academy of Sleep Medicine and the Sleep Research Society have determined that adults require ≥7 hours of sleep per day to promote optimal health (*1*). Short sleep duration (<7 hours per day) has been linked to adverse health outcomes including cardiovascular disease, obesity, diabetes, depression, and anxiety, as well as safety issues related to drowsy driving and injuries (*1*,*2*). Additional research has found that sleep duration varies by characteristics such as race, education, marital status, obesity, and cigarette smoking (*3*). Work-related factors such as job stress, work hours, shift work, and physically demanding work have been found to be associated with sleep duration and quality (*4*–*6*). All of these work factors vary by industry and occupation of employment, and the prevalence of short sleep duration has been shown to vary by broad industry and occupation category (*7*). To provide updated and more detailed information about which occupation groups have the highest prevalences of short sleep duration, CDC analyzed data from currently employed adults surveyed for the 2013 and 2014 Behavioral Risk Factor Surveillance System (BRFSS) in 29 states. Among 22 major occupation groups, the highest prevalences of short sleep duration were among workers in the following five groups: Production (42.9%), Healthcare Support (40.1%), Healthcare Practitioners and Technical (40.0%), Food Preparation and Serving-Related (39.8%), and Protective Service (39.2%). The significant differences among occupation groups in the prevalence of short sleep duration suggest that work-related factors should be further evaluated as they might relate to sleep.

BRFSS is an annual, random-digit–dialed telephone survey of noninstitutionalized, U.S. civilian residents aged ≥18 years. It is conducted by U.S. states and territories to gather data on health-related risk behaviors, chronic illnesses and conditions, and use of health-related services.* The BRFSS questionnaire is composed of a set of core questions that are asked by all states; in addition, states may choose from optional modules on specific subjects or include state-added questions. Twenty-nine states^†^ administered the optional industry and occupation module in 2013 or 2014. Response rates for BRFSS are calculated based on American Association of Public Opinion Research guidelines. The median response rate for all states, territories, and the District of Columbia was 46.4% in 2013 and 47.0% in 2014, whereas the response rates for states included in analyses ranged from 31.1% to 59.2% in 2013 and from 33.0% to 57.6% in 2014.^§^

To determine occupation, BRFSS participants who were employed for wages, self-employed, or out of work for <1 year were asked, “What kind of work do you do?” Participants’ responses were recorded as free text and later coded to one of the 574 U.S. Bureau of Census (2002) occupation numeric codes^¶^ by an auto-coding system or computer-assisted human coders. Because of the difficulty in reporting results for such a large number of occupations, and to protect participants’ privacy, the 574 Bureau of Census codes were grouped into 93 two-digit detailed occupation groups used by CDC’s National Center for Health Statistics to code occupations for the National Health Interview Survey,** and these detailed groups were collapsed into the 22 two-digit Standard Occupational Classification System major occupation groups created by the Bureau of Labor Statistics.^††^ Respondents also were asked, “On average, how many hours of sleep do you get in a 24-hour period?” Responses to this question were dichotomized into ≥7 hours of sleep (sufficient sleep) and <7 hours of sleep (short sleep duration).

Among the 412,829 BRFSS participants in 2013 and 2014, a total of 207,143 (50.2%) were currently employed for wages, self-employed, or out of work for <1 year and were considered for analyses. After excluding 523 respondents (0.3%) who were on active military duty, 249 (0.1%) who were unpaid or retired workers, and 26,750 (12.9%) with insufficient or missing information necessary for occupational coding, the final sample for analyses totaled 179,621 (86.7% of the currently employed respondents). Prevalence of short sleep duration was calculated by the 22 major and 93 detailed occupation groups and adjusted for the following characteristics: age group (18–34 years, 35–44 years, 45–54 years, 55–64 years, and ≥65 years); sex (male or female); race/ethnicity (non-Hispanic white, non-Hispanic black, Hispanic, and non-Hispanic other race or multiracial); marital status (married/member of an unmarried couple or divorced/widowed/separated/never married); and education level (less than high school diploma, graduated high school, some college, or college graduate). The adjusted prevalence estimates were obtained using logistic regression. F tests were used as a measure of association to determine statistical significance of the variables. All analyses were weighted to account for the survey design unless otherwise noted.

Overall, 36.5% of currently employed adults reported short sleep duration. The prevalence of short sleep duration among persons in the three youngest age groups was similar (18–34 years [37.7%], 35–44 years [37.6%], and 45–54 years [37.4%]) and lower among persons in the two oldest age groups (45-64 years [33.8%] and ≥65 years [29.2%]). Among persons categorized by other characteristics, the highest prevalences were reported by men (37.5%), non-Hispanic blacks (48.5%), persons with some college education (40.0%), and persons who were divorced, widowed, separated or never married (39.5%) (Table 1).

**TABLE 1:** Prevalence of short sleep duration (<7 hours of sleep per day) among currently employed adults, by selected characteristics — Behavioral Risk Factor Surveillance System, 29 states, 2013–2014

Characteristic	Unweighted no.	Weighted % of total sample population (95% CI)	Prevalence of short sleep % (95% CI)
**Age group (yrs)**			
18–34	41,326	32.8 (32.3–33.4)	37.7 (36.6–38.7)
35–44	38,258	22.2 (21.8–22.7)	37.6 (36.4–38.8)
45–54	52,189	23.4 (23.0–23.9)	37.4 (36.4–38.5)
55–64	54,089	16.5 (16.2–16.9)	33.8 (32.7–34.9)
≥65	21,281	5.0 (4.8–5.2)	29.2 (27.3–31.2)
**Sex**			
Men	98,868	54.8 (54.3–55.3)	37.5 (36.7–38.2)
Women	108,275	45.2 (44.7–45.7)	35.4 (34.6–36.1)
**Race/Ethnicity**			
White, non-Hispanic	165,130	63.4 (62.8–63.9)	33.5 (32.9–34.0)
Black, non-Hispanic	13,523	11.1 (10.7–11.5)	48.5 (46.8–50.2)
Hispanic	14,656	16.8 (16.4–17.3)	37.8 (36.1–39.5)
Other race or multiracial, non-Hispanic	10,633	8.7 (8.3–9.1)	39.9 (37.2–42.5)
**Education**			
Less than high school diploma	8,863	10.7 (10.3–11.2)	37.4 (35.3–39.5)
Graduated high school	48,818	24.9 (24.5–25.4)	38.9 (37.8–39.9)
Some college	57,291	31.0 (30.5–31.5)	40.0 (39.0–41.1)
College graduate	91,704	33.3 (32.8–33.8)	31.3 (30.6–32.1)
**Marital status**			
Married/Member of an unmarried couple	130,990	60.8 (60.3–61.4)	34.7 (34.0–35.3)
Divorced/Widowed/Separated/Never married	74,856	39.2 (38.6–39.7)	39.5 (38.6–40.4)
**State of residence**			
California	3,706	18.0 (17.6–18.4)	37.3 (35.3–39.4)
Colorado	4,590	2.8 (2.8–2.9)	29.2 (27.4–31.0)
Connecticut	4,257	1.9 (1.8–1.9)	36.3 (34.4–38.3)
Florida	12,982	9.1 (8.9–9.3)	38.9 (37.2–38.3)
Georgia	2,857	4.8 (4.7–4.9)	38.8 (36.5–41.1)
Idaho	2,628	0.8 (0.7–0.8)	32.2 (29.7–34.8)
Illinois	5,403	6.5 (6.4–6.6)	35.9 (34.2–37.6)
Iowa	4,370	1.7 (1.6–1.7)	32.4 (30.6–34.1)
Louisiana	5,449	2.1 (2.1–2.2)	37.4 (35.5–39.2)
Maryland	13,210	3.2 (3.2–3.3)	40.6 (39.2–42.0)
Massachusetts	15,405	3.5 (3.5–3.6)	35.2 (34.1–36.4)
Michigan	9,811	4.6 (4.5–4.7)	40.3 (39.1–41.6)
Minnesota	18,291	3.1 (3.0–3.1)	31.6 (30.5–32.6)
Mississippi	4,735	1.3 (1.3–1.4)	35.9 (34.0–37.9)
Montana	8,729	0.5 (0.5–0.5)	31.1 (29.8–32.5)
Nebraska	11,011	1.0 (1.0–1.0)	32.5 (31.1–34.0)
New Hampshire	6,628	0.7 (0.7–0.7)	32.5 (31.0–34.1)
New Jersey	4,805	4.7 (4.5–4.8)	39.6 (37.5–41.8)
New Mexico	8,571	0.9 (0.9–1.0)	33.1 (31.7–34.6)
New York	3,826	9.8 (9.6–10.1)	40.1 (38.0–42.3)
North Carolina	3,408	4.8 (4.7–4.9)	32.7 (30.8–34.5)
North Dakota	9,082	0.4 (0.4–0.4)	32.0 (30.6–33.5)
Oregon	4,966	1.8 (1.8–1.9)	31.8 (30.1–33.5)
Tennessee	2,073	3.1 (3.0–3.2)	36.4 (33.5–39.3)
Utah	15,806	1.5 (1.4–1.5)	33.9 (32.9–34.8)
Vermont	3,945	0.4 (0.4–0.4)	29.7 (28.0–31.4)
Washington	10,111	3.5 (3.4–3.6)	33.6 (32.4–34.9)
Wisconsin	3,410	2.9 (2.9–3.0)	32.9 (30.5–35.3)
Wyoming	3,078	0.3 (0.3–0.3)	29.2 (27.1–31.4)
**Overall**	**207,143**	**56.3 (55.9–56.7)**	**36.5 (36.0–37.1)**

**Abbreviation:** CI = confidence interval.

Among the 22 major occupation groups, the highest prevalences of short sleep duration were among workers in the following five groups: Production (42.9%), Healthcare Support (40.1%), Healthcare Practitioners and Technical (40.0%), Food Preparation and Serving-Related (39.8%), and Protective Service (39.2%). The two major occupation groups with the lowest prevalence of short sleep duration were Education, Training, and Library and Farming, Fishing, and Forestry (both 31.3%) (Table 2).

**TABLE 2:** Prevalence of short sleep duration (<7 hours of sleep per day) among currently employed adults, by Standard Occupational Classification (SOC) System major occupation groups and detailed occupation groups* — Behavioral Risk Factor Surveillance System (BRFSS), 29 states, 2013–2014

Major occupation group (SOC code)/Detailed occupation group	Unweighted no.	Unadjusted % (95% CI)	Adjusted^†^ % (95% CI)	CV for adjusted %
**Production (51)**	**7,605**	**44.6 (42.0–47.2)**	**42.9 (40.3–45.4)**	**0.03**
Printing workers	216	52.3 (38.1–66.6)	50.9 (37.1–64.6)	0.14
Plant and system operators	503	52.3 (40.4–64.3)	49.6 (38.7–60.5)	0.11
Supervisors, production workers	546	50.3 (39.9–60.8)	48.9 (39.0–58.9)	0.10
Other production occupations	2,671	47.1 (42.9–51.3)	45.6 (41.5–49.8)	0.05
Metal workers and plastic workers	1,478	45.3 (40.5–50.2)	44.0 (39.2–49.0)	0.06
Woodworkers	199	40.3 (24.2–56.4)	39.2 (25.9–54.4)	0.19
Assemblers and fabricators	811	39.4 (32.2–46.6)	36.8 (29.9–44.2)	0.10
Food processing workers	543	37.9 (28.7–47.1)	35.9 (27.5–45.3)	0.13
Textile, apparel, and furnishings workers	638	34.6 (24.7–44.6)	34.2 (24.9–44.8)	0.15
**Healthcare Support (31)**	**4,328**	**42.7 (39.4–46.1)**	**40.1 (36.7–43.5)**	**0.04**
Nursing, psychiatric, and home health aides	2,484	47.8 (43.3–52.3)	43.3 (38.9–47.8)	0.05
Other healthcare support occupations	1,732	35.8 (30.6–41.0)	35.7 (30.5–41.3)	0.08
Occupational and physical therapist assistants and aides	112	30.5 (15.8–45.1)	32.8 (19.7–49.4)	0.24
**Healthcare Practitioners and Technical (29)**	**14,975**	**38.1 (35.9–40.2)**	**40.0 (37.8–42.2)**	**0.03**
Health technologists and technicians	3,218	41.0 (37.2–44.9)	40.4 (36.7–44.3)	0.05
Health diagnosing and treating practitioners	11,589	37.2 (34.7–39.7)	39.7 (37.0–42.4)	0.04
Other healthcare practitioners and technical occupations	168	33.4 (15.6–51.2)	35.1 (21.0–52.6)	0.24
**Food Preparation and Serving-Related (35)**	**5,413**	**42.4 (39.1–45.6)**	**39.8 (36.6–43.0)**	**0.04**
Supervisors, food preparation, and serving workers	910	53.1 (44.8–61.4)	48.9 (40.6–57.3)	0.09
Cooks and food preparation workers	2,162	44.3 (38.9–49.7)	41.4 (36.3–46.8)	0.07
Food and beverage serving workers	1,876	37.4 (32.4–42.3)	36.1 (31.4–41.1)	0.07
Other food preparation and serving related workers	465	33.0 (23.0–43.1)	30.8 (21.7–41.6)	0.17
**Protective Service (33)**	**3,462**	**42.4 (38.6–46.2)**	**39.2 (35.6–43.0)**	**0.05**
Firefighting and prevention workers	534	48.0 (39.0–56.9)	45.8 (37.1–54.7)	0.10
Law enforcement officers	1,591	42.2 (36.8–47.7)	39.8 (34.6–45.3)	0.07
Other protective service workers	1,129	41.9 (35.0–48.7)	37.7 (31.4–44.4)	0.09
First-line supervisors/managers, protective service workers	208	26.4 (13.2–39.6)	23.7 (13.0–39.3)	0.28
**Transportation and Material Moving (53)**	**8,014**	**42.3 (39.7–44.9)**	**39.1 (36.6–41.7)**	**0.03**
Other transportation workers	138	56.5 (39.4–73.7)	54.0 (35.9–71.2)	0.17
Rail transportation workers	227	54.5 (39.2–69.8)	52.7 (37.4–67.4)	0.15
Supervisors, transportation and material moving employees	141	48.0 (29.1–66.9)	43.3 (26.0–62.4)	0.22
Material moving workers	2,337	44.2 (39.5–48.8)	40.5 (36.0–45.1)	0.06
Motor vehicle operators	4,823	41.5 (38.0–44.9)	38.5 (35.2–41.9)	0.04
Water transportation workers	99	30.0 (14.8–45.2)	31.5 (18.4–48.4)	0.25
Air transportation workers	249	20.6 (11.6–29.7)	21.4 (13.3–32.8)	0.23
**Personal Care and Service (39)**	**5,907**	**38.9 (35.3–42.5)**	**37.5 (34.0–41.1)**	**0.05**
Supervisors, personal care and service workers	153	32.8 (14.5–51.0)	34.3 (17.7–55.9)	0.30
Animal care and service workers	289	34.1 (19.2–48.9)	35.3 (22.0–51.3)	0.22
Entertainment attendants and related workers	219	51.7 (25.1–78.3)	48.2 (27.1–69.9)	0.24
Personal appearance workers	1,114	34.1 (27.3–40.9)	31.7 (25.4–38.9)	0.11
Transportation, tourism, and lodging attendants	236	41.7 (27.6–55.7)	36.4 (25.1–49.4)	0.17
Other personal care and service workers	3,876	39.4 (35.3–43.5)	38.5 (34.4–42.7)	0.05
Funeral service workers	20	—^§^	—	0.18
**Installation, Maintenance, and Repair (49)**	**5,328**	**38.4 (35.2–41.5)**	**36.6 (33.5–39.8)**	**0.04**
Other installation, maintenance, and repair occupations	1,946	39.9 (34.7–45.2)	38.7 (33.6–44.1)	0.07
Electrical and electronic equipment mechanics, installers, and repairers	713	39.1 (30.4–47.9)	36.6 (28.4–45.7)	0.12
Vehicle and mobile equipment mechanics, installers, and repairers	2,431	37.7 (33.1–42.4)	36.0 (31.6–40.7)	0.06
Supervisors of installation, maintenance, and repair workers	238	27.2 (16.1–38.3)	27.5 (18.0–39.6)	0.20
**Office and Administrative Support (43)**	**21,406**	**36.6 (34.9–38.3)**	**36.5 (34.8–38.3)**	**0.02**
Communications equipment operators	109	59.0 (43.1–74.9)	58.2 (42.6–72.3)	0.13
Material recording, scheduling, dispatching, and distribution workers	2,584	46.2 (41.1–51.3)	44.6 (39.5–49.9)	0.06
Other office and administrative support workers	5,325	35.7 (32.0–39.3)	36.0 (32.3–39.7)	0.05
Information and record clerks	4,279	36.9 (33.5–40.3)	35.9 (32.5–39.3)	0.05
Financial clerks	3,539	34.8 (30.3–39.3)	35.3 (30.9–40.0)	0.07
Supervisors, office and administrative support workers	2,139	31.6 (27.4–35.9)	33.3 (29.1–37.8)	0.07
Secretaries and administrative assistants	3,431	31.7 (28.1–35.3)	32.4 (29.0–36.1)	0.06
**Business and Financial Operations (13)**	**7,811**	**33.9 (31.1–36.8)**	**36.1 (33.3–39.0)**	**0.04**
Business operations specialists	3,734	34.7 (30.5–39.0)	36.0 (32.2–40.0)	0.06
Financial specialists	4,077	33.1 (29.2–36.9)	36.0 (32.2–40.0)	0.06
**Building and Grounds Cleaning and Maintenance (37)**	**6,265**	**38.0 (35.1–40.9)**	**36.0 (33.2–39.0)**	**0.04**
Supervisors, building and grounds cleaning and maintenance workers	415	42.4 (33.7–51.2)	41.2 (33.3–49.6)	0.10
Building cleaning and pest control workers	4,750	40.2 (36.8–43.6)	38.2 (34.8–41.6)	0.04
Grounds maintenance workers	1,100	30.4 (24.1–36.6)	28.8 (23.1–35.3)	0.11
**Arts, Design, Entertainment, Sports, and Media (27)**	**4,124**	**33.5 (28.7–38.3)**	**35.5 (31.1–40.2)**	**0.06**
Art and design workers	1,569	38.0 (28.6–47.4)	39.0 (31.0–47.7)	0.11
Entertainers and performers, sports and related workers	769	33.7 (23.8–43.6)	34.8 (26.1–44.6)	0.14
Media and communication workers	1,392	29.3 (23.7–34.8)	33.6 (28.1–39.6)	0.09
Media and communication equipment workers	394	28.4 (17.6–39.2)	29.3 (19.6–41.3)	0.19
**Management (11)**	**21,808**	**33.8 (32.1–35.4)**	**35.4 (33.7–37.2)**	**0.03**
Chief, executives; general and operations managers; legislators	2,529	33.4 (29.4–37.4)	36.3 (32.2–40.6)	0.06
Operations specialties managers	3,167	34.1 (30.3–37.8)	35.6 (31.8–39.6)	0.06
Other management occupations	14,795	34.0 (31.9–36.2)	35.3 (33.0–37.5)	0.03
Advertising, marketing, promotions, public relations, and sales managers	1,317	31.7 (25.8–37.5)	34.1 (28.4–40.3)	0.09
**Legal (23)**	**2,694**	**31.1 (27.3–34.8)**	**34.5 (30.6–38.5)**	**0.06**
Legal support workers	758	35.9 (28.5–43.2)	37.5 (30.4–45.1)	0.10
Lawyers, judges, and related workers	1,936	29.1 (24.7–33.5)	32.9 (28.3–37.7)	0.07
**Construction and Extraction (47)**	**9,208**	**36.1 (33.8–38.3)**	**34.5 (32.2–36.9)**	**0.03**
Extraction workers	575	46.0 (36.7–55.3)	45.3 (36.3–54.7)	0.10
Construction trades workers	6,975	36.2 (33.6–38.7)	34.6 (32.0–37.3)	0.04
Other construction and related workers	458	34.3 (23.6–45.0)	34.5 (24.4–46.2)	0.16
Supervisors, construction and extraction workers	1,184	34.4 (28.8–40.1)	34.2 (28.8–40.1)	0.08
Helpers, constructions trades	16	—	—	0.89
**Sales and Related (41)**	**16,526**	**34.9 (33.0–36.7)**	**34.4 (32.6–36.3)**	**0.03**
Supervisors, sales workers	3,332	36.0 (31.8–40.2)	36.0 (32.0–40.2)	0.06
Sales representatives, services	2,214	33.7 (28.9–38.5)	35.4 (30.7–40.4)	0.07
Retail sales workers	7,243	36.3 (33.4–39.1)	34.4 (31.7–37.3)	0.04
Other sales and related workers	2,408	32.4 (27.3–37.4)	33.5 (28.6–38.8)	0.08
Sales representatives, wholesale and manufacturing	1,329	29.1 (23.8–34.5)	30.3 (25.1–36.1)	0.09
**Architecture and Engineering (17)**	4,886	32.6 (29.3–35.8)	34.3 (31.0–37.9)	0.05
Drafters, engineering, and mapping technicians	847	42.0 (34.1–49.9)	40.5 (33.0–48.5)	0.10
Architects, surveyors, and cartographers	432	33.8 (21.6–46.0)	36.2 (24.6–49.7)	0.18
Engineers	3,607	29.8 (26.2–33.5)	32.2 (28.4–36.3)	0.06
**Computer and Mathematical (15)**	**5,591**	**33.3 (30.5–36.1)**	**33.8 (31.1–36.7)**	**0.04**
Mathematical science occupations	278	36.8 (25.6–48.0)	38.1 (27.4–50.2)	0.15
Computer specialists	5,313	33.2 (30.3–36.1)	33.6 (30.7–36.6)	0.04
**Life, Physical, and Social Science (19)**	**3,265**	**30.2 (26.5–34.0)**	**33.6 (29.7–37.7)**	**0.06**
Life, physical, and social science technicians	552	41.3 (32.0–50.6)	41.8 (32.9–51.2)	0.11
Physical scientists	929	28.8 (22.6–35.0)	32.4 (25.9–39.6)	0.11
Social scientists and related workers	970	27.9 (20.3–35.4)	32.3 (24.8–40.9)	0.13
Life scientists	814	23.8 (17.9–29.8)	26.8 (20.9–33.6)	0.12
**Community and Social Services (21)**	**4,224**	**31.3 (26.9–35.7)**	**32.2 (27.7–37.1)**	**0.07**
Counselors, social workers, and other community and social service specialists	3,322	33.6 (28.5–38.7)	34.0 (28.8–39.7)	0.08
Religious workers	902	20.5 (15.2–25.7)	22.4 (17.2–28.6)	0.13
**Education, Training, and Library (25)**	**15,249**	**27.9 (26.2–29.7)**	**31.3 (29.2–33.4)**	**0.04**
Postsecondary teachers	2,351	21.8 (18.4–25.3)	25.4 (21.7–29.4)	0.08
Primary, secondary, and special education school workers	9,806	29.0 (26.7–31.2)	32.5 (29.9–35.1)	0.04
Other teachers and instructors	834	23.6 (17.1–30.2)	25.2 (19.0–32.7)	0.14
Librarians, curators, and archivists	628	26.2 (18.5–33.9)	30.3 (22.9–38.8)	0.14
Other education, training, and library occupations	1,630	31.6 (25.8–37.4)	32.5 (26.8–38.8)	0.10
**Farming, Fishing, and Forestry (45)**	**1,532**	**32.1 (24.5–38.7)**	**31.3 (25.0–38.4)**	**0.11**
Fishing and hunting workers	82	35.1 (16.0–54.2)	36.6 (20.4–56.5)	0.26
Agricultural workers	1,210	31.0 (23.6–38.4)	30.2 (23.1–38.3)	0.13
Forest, conservations, and logging workers	179	—	—	0.31
Supervisors, farming, fishing, and forestry workers	61	—	—	0.41
**All occupation groups**	**179,621**	**36.5 (35.9–37.1)**	—	—

**Abbreviations:** CI = confidence interval; CV = coefficient of variation.

* To determine occupation, Behavioral Risk Factor Surveillance System participants who were employed for wages, self-employed, or out of work for <1 year were asked, “What kind of work do you do?” Participants’ responses were recorded as free text and later coded to one of the 574 U.S. Bureau of Census (2002) occupation numeric codes by an auto-coding system or computer-assisted human coders. Because of the difficulty in reporting results for such a large number of occupations, and to protect participants’ privacy, the 574 Bureau of Census codes were grouped into 93 two-digit detailed occupation groups used by CDC’s National Center for Health Statistics to code occupations for the National Health Interview Survey, and these detailed groups were collapsed into the 22 two-digit SOC major occupation groups created by the Bureau of Labor Statistics. Respondents also were asked, “On average, how many hours of sleep do you get in a 24-hour period?” Responses to this question were dichotomized into ≥7 hours of sleep (sufficient sleep) and <7 hours of sleep (short sleep duration).

^†^ Adjusted by sex, race/ethnicity, marital status, age group, and education level.

^§^ Estimates were suppressed because they did not meet the statistical reliability standards of BRFSS (i.e., cell size was <50 participants or CV >0.30).

Within the Protective Service major occupation group, the highest prevalence of short sleep duration was reported in the detailed group of firefighting and prevention workers (45.8%). Within the Healthcare Support major group, the highest prevalences were reported in the detailed group of nursing, psychiatric, and home health aides (43.3%). Among all major occupation groups, the detailed groups with the highest prevalences of short sleep duration were communications equipment operators (58.2%), other transportation workers (54.0%), and rail transportation workers (52.7%). The detailed groups with the lowest prevalences of short sleep duration were air transportation workers (21.4%) and religious workers (22.4%) (Table 2).

For the 29 states, the weighted percentage of currently employed adults in any of the five major occupation groups with the highest prevalence of short sleep duration also was calculated. Among the states, the percentage of currently employed adults working in any of the five major occupation groups with the highest prevalence of short sleep duration ranged from 17.6% (Wyoming) to 26.8% (Mississippi) (Table 3).

**TABLE 3:** Weighted percentage of currently employed adults in the five major occupation groups of the Standard Occupational Classification System with the highest prevalence of short sleep duration (<7 hours of sleep per day), by state — Behavioral Risk Factor Surveillance System, 29 states, 2013–2014

State	Top five major occupation groups					
	Production	Healthcare Support	Healthcare Practitioners and Technical	Food Preparation and Serving-Related	Protective Service	Any of the top five major occupation groups
Mississippi	7.7	2.5	8.2	5.2	3.2	26.8
Tennessee	8.0	2.6	6.6	5.6	2.5	25.3
Wisconsin	10.1	3.1	6.2	3.9	2.0	25.3
Louisiana	4.8	3.3	7.7	6.2	3.0	25.0
Michigan	8.1	3.2	7.4	4.2	1.9	24.8
North Carolina	5.7	3.1	7.2	4.2	2.5	22.7
Iowa	7.5	3.2	7.0	3.1	1.5	22.3
New York	3.9	3.1	8.0	4.8	2.5	22.3
Illinois	6.5	3.0	6.3	4.4	1.9	22.1
Minnesota	6.1	2.8	8.1	3.9	1.2	22.1
Oregon	5.8	2.5	6.8	4.1	2.5	21.7
Massachusetts	3.7	2.8	9.6	3.6	1.8	21.5
Florida	3.2	3.0	6.8	5.4	3.1	21.5
Vermont	6.0	2.4	6.9	4.5	1.6	21.4
Connecticut	4.7	3.0	8.0	3.4	2.3	21.4
Nebraska	6.0	2.6	7.3	3.8	1.6	21.3
North Dakota	5.0	2.8	6.8	4.3	1.9	20.8
Idaho	5.9	2.7	5.1	4.4	2.4	20.5
New Hampshire	4.7	3.1	7.6	3.1	1.7	20.2
Montana	4.8	2.5	5.8	4.4	2.4	19.9
California	5.1	1.7	5.4	5.0	2.1	19.3
New Jersey	3.2	2.9	7.6	2.4	3.2	19.3
Washington	4.6	2.6	6.0	4.2	1.8	19.2
New Mexico	3.6	2.6	6.2	3.8	2.9	19.1
Georgia	5.2	1.6	5.6	4.6	1.8	18.8
Maryland	1.9	2.3	6.9	4.2	3.5	18.8
Utah	5.2	2.4	5.6	3.3	2.2	18.7
Colorado	3.6	2.2	6.0	4.2	2.5	18.5
Wyoming	5.2	1.7	5.2	3.2	2.3	17.6

## Discussion

This study is the first to evaluate short sleep duration by 93 detailed occupation groups and at a multistate level. A previous study using National Health Interview Survey data found that within certain industries, production and transportation and material moving occupations had among the highest prevalences of short sleep duration, a finding that is consistent with the results of this study (*7*). Previous studies have shown that shift workers are more likely to experience disturbed sleep and excessive sleepiness, and to report a significantly higher prevalence of short sleep duration compared with day workers (*6*,*8*). Shift work negatively influences health, by affecting the natural circadian rhythm, leading to irregularities in the sleep-wake cycle (*8*). The five major occupation groups with the highest prevalence of short sleep duration (Production, Healthcare Support, Healthcare Practitioners and Technical, Food Preparation and Serving-Related, and Protective Service) also have some of the highest prevalence rates of alternative shift work, ranging from >35% of Healthcare Practitioners and Technical workers to >50% of Food Preparation and Serving-Related workers (*9*).

Respondents working in detailed occupation groups within the major occupation group of Transportation and Material Moving reported a wide range in prevalences of short sleep duration, from air transportation workers (21%) to other transportation workers (54%). In 2011, the Federal Aviation Administration overhauled commercial airline pilot scheduling to ensure that pilots are rested before flying; this might account for the low prevalence of short sleep duration among air transportation workers.^§§^ In contrast, 53% of rail transportation workers reported short sleep duration. Although the Railroad Safety Improvement Act of 2008 mandated changes to the limitations on the number of hours railroad employees work, compliance with the bill is not required until 2018.^¶¶^ Shift work and existing occupational regulations likely are important factors to consider regarding the results of this study.

The findings in this report are subject to at least four limitations. First, because BRFSS data are cross-sectional, it is not possible to determine temporal relationships. Second, BRFSS data are self-reported, and are therefore subject to recall and social desirability biases. Among certain occupations where sleep duration has been an issue and hours might be specified by regulation (e.g., transportation), there might be a greater sensitivity to this question and a bias toward reporting sufficient sleep. Third, because the data came from 29 states, the results might not be representative of the national currently employed population. Finally, misclassification of occupation by respondents, interviewers or coders, although likely rare, is possible.

Short sleep duration among the U.S. working population has been estimated to result in a $411 billion dollar annual cost to the economy, equivalent to 2.28% of the country’s gross domestic product (*10*). In addition, among employed persons, 1.2 million working days are lost in the United States each year because of sleep deprivation. It is estimated that if persons who sleep <6 hours per day began sleeping for 6–7 hours per day, approximately $226 billion could be added to the U.S. economy (*10*). A goal of *Healthy People 2020* is to “increase public knowledge of how adequate sleep and treatment of sleep disorders improve health, productivity, wellness, quality of life, and safety on roads and in the workplace,” with a specific objective to increase the proportion of adults getting sufficient sleep.***

CDC’s National Institute for Occupational Safety and Health has developed educational resources on shift work and long working hours for managers, workers, and the public.^†††^ The materials include interactive training for nurses, emergency responders, and truck drivers, as well as information for aviation and railroad employees, methods for improving shiftwork schedules, and individual coping strategies. Time at work continues to increase, with the United States having the longest annual working hours among all wealthy industrialized countries (*7*). Job characteristics, such as schedules, stress, and physical output, should be evaluated in an effort to improve worker sleep duration and overall health.

**Summary**

**What is already known about this topic?**

Shift work and other work factors influence sleep duration and sleep quality, which have a direct effect on worker health and safety. Previous research has found that workers in production, health care, protective service, transportation and material moving, and food preparation and serving-related fields are more likely to be shift workers. In addition, production and transportation and material moving occupations have been associated with higher prevalences of short sleep duration.

**What is added by this report?**

Analysis of 2013 and 2014 Behavioral Risk Factor Surveillance System data conducted to examine 93 detailed occupation groups in 29 states found that the prevalence of <7 hours of sleep per day (short sleep duration) ranged from 21.4% among air transportation workers to 58.2% among communications equipment workers. The percentage of workers employed in at least one of the five occupations with the highest prevalence of short sleep duration ranged from 17.6% in Wyoming to 26.8% in Mississippi.

**What are the implications for public health practice?**

Significant differences were found in the prevalence of short sleep duration among occupation groups. Workers in occupations with high prevalences of short sleep duration might be most at risk for sleep-related accidents and adverse health outcomes associated with short sleep duration. Work-related factors should be further evaluated in the context of short sleep duration to guide prevention efforts.

## Footnotes

* https://www.cdc.gov/brfss/annual_data/2013/pdf/overview_2013.pdf and https://www.cdc.gov/brfss/annual_data/2014/pdf/overview_2014.pdf.

† States providing data in 2013 and 2014: Illinois, Louisiana, Maryland, Massachusetts, Michigan, Minnesota, Mississippi, Montana, Nebraska, New Hampshire, New Jersey, New Mexico, New York, North Dakota, Oregon, Utah, and Washington. States contributing data only in 2013: California, Florida, Wisconsin, and Wyoming. States contributing data only in 2014: Colorado, Connecticut, Georgia, Idaho, Iowa, North Carolina, Tennessee, and Vermont. Washington and Wyoming’s 2013 industry and occupation data are from state-added questions, provided with permission of the two states’ BRFSS coordinators.

§ https://www.cdc.gov/brfss/annual_data/2013/pdf/2013_dqr.pdf and https://www.cdc.gov/brfss/annual_data/2014/pdf/2014_dqr.pdf.

¶ https://www.census.gov/people/io/methodology/.

**
ftp://ftp.cdc.gov/pub/Health_Statistics/NCHS/Dataset_Documentation/NHIS/2007/srvydesc.pdf .

†† https://www.bls.gov/soc/major_groups.htm.

§§
https://www.faa.gov/news/fact_sheets/news_story.cfm?newsId=13273 .

¶¶ https://www.fra.dot.gov/eLib/details/L04320.

***
https://www.healthypeople.gov/2020/topics-objectives/topic/sleep-health .

†††
https://www.cdc.gov/niosh/topics/workschedules/education.html .
